# Family, social and cultural determinants of long-lasting insecticidal net (LLIN) use in Madagascar: secondary analysis of three qualitative studies focused on children aged 5–15 years

**DOI:** 10.1186/s12936-021-03705-2

**Published:** 2021-03-26

**Authors:** Ammy Fiadanana Njatosoa, Chiarella Mattern, Dolorès Pourette, Thomas Kesteman, Elliot Rakotomanana, Bakoly Rahaivondrafahitra, Mauricette Andriamananjara, Aina Harimanana, Jocelyn Razafindrakoto, Emma Raboanary, Andry Andrianasolo, Christophe Rogier

**Affiliations:** 1 Groupe Santé & Sciences Sociales, Unité Épidémiologie Et de Recherche Clinique, Institut Pasteur de Madagascar, Antananarivo, Madagascar; 2 IRD, Ceped (Institut de Recherche Pour Le Développement, Université de Paris, INSERM), Paris, France; 3 Unité de Recherche Sur Le Paludisme, Institut Pasteur de Madagascar, Antananarivo, Madagascar; 4 Oxford University Clinical Research Unit, Hanoi, Vietnam; 5 Population Services International Madagascar, Antananarivo, Madagascar; 6 Programme National de Lutte Contre Le Paludisme, Ministère de La Santé Publique, Antananarivo, Madagascar; 7 Unité Épidémiologie Et de Recherche Clinique, Institut Pasteur de Madagascar, Antananarivo, Madagascar; 8 President’s Malaria Initiative, Antananarivo, Madagascar; 9 Institut International Des Sciences Sociales, Antananarivo, Madagascar; 10 Institut Pasteur de Madagascar, Antananarivo, Madagascar; 11 Primum Vitare, Paris, France

**Keywords:** Malaria, LLIN use, Children over five, Sociocultural factors, Madagascar

## Abstract

**Background:**

Although it is accepted that long-lasting insecticidal net (LLIN) use is an effective means to prevent malaria, children aged 5 to 15 years do not appear to be sufficiently protected in Madagascar; the malaria prevalence is highest in this age group. The purpose of this research is to summarize recent qualitative studies describing LLIN use among the Malagasy people with a focus on children aged 5–15 years.

**Methods:**

Qualitative data from three studies on malaria conducted between 2012 and 2016 in 10 districts of Madagascar were analysed. These studies cover all malaria epidemiological profiles and 10 of the 18 existing ethnic groups in Madagascar. A thematic analysis was conducted on the collected data from semi-structured interviews, direct observation data, and informal interviews.

**Results:**

A total of 192 semi-structured interviews were conducted. LLINs are generally perceived positively because they protect the health and well-being of users. However, regional representations of mosquito nets may contribute to LLIN lower use by children over 5 years of age including the association between married status and LLIN use, which leads to the refusal of unmarried young men to sleep under LLINs; the custom of covering the dead with a mosquito net, which leads to fear of LLIN use; and taboos governing sleeping spaces for siblings of opposite sexes, which leads to LLIN shortages in households. Children under 5 years of age are known to be the most vulnerable age group for acquiring malaria and, therefore, are prioritized for LLIN use when there are limited supplies in households. In contrast, children over 5 years of age, who are perceived to be at less risk for malaria, often sleep without LLINs.

**Conclusions:**

Perceptions, social practices and regional beliefs regarding LLINs and vulnerability to malaria contribute to the nonuse of LLINs among children over 5 years of age in Madagascar. Modifying LLIN policies to account for these factors may increase LLIN use in this age group and reduce disease burden.

## Background

Insecticide-treated nets have been demonstrated to reduce malaria infections in a variety of settings [1]. Hill et al*.* [2] qualified it as “the most powerful malaria control tool to be developed since the advent of indoor residual spraying and chloroquine in the 1940s”. In Madagascar, Kesteman et al*.* [3] demonstrated that the protective effectiveness of a long-lasting insecticidal net (LLIN) can reach 72%. LLINs have made it possible to avoid more than 100,000 clinical cases of malaria each year [3]. In the southeast of Madagascar, the same author showed that nighttime LLIN use was significantly associated with lower parasite prevalence [4]. In areas with high LLIN coverage, people who do not sleep under a LLIN are at lower risk of malaria because of the reduction in overall malaria transmission in the area [5, 6].

Due to the combined efforts of financial partners and the Ministry of Public Health of Madagascar, a total of 17,858,084 LLINs were distributed throughout the island between 2009 and 2013 [7]. For the total population of 22,961,253 in 2013 [8], the index of this universal coverage would be 1.28 persons per LLIN. However, Madagascar is one of the 7 countries in the world where the incidence of malaria and the mortality rate related to malaria have increased by 20% or more between 2010 and 2015 [9, 10]. The age group most affected consists of children over 5 years of age. In 2012, Kesteman et al*.* [11] showed that children aged 5 to 15 years were twice as likely to test positive for *Plasmodium falciparum* as were children under 5 years of age. Children aged 5–19 years account for almost two-thirds (57.8%) of those who test positive on the Rapid Diagnostic Test (RDT) for malaria [11].

The importance of malaria morbidity among 5- to 15-year-olds in Madagascar has already been considered by the Ministry of Health through the National Strategic Plan of 2018–2022, which envisages extending community malaria management to this age group through the Integrated Management of Childhood Illness [12]. Although the effectiveness of this policy has not been proven, it offers hope for 5- to 15-year-olds and has already led to a significant reduction in under-five mortality worldwide through precise diagnoses of the main childhood diseases, the provision of appropriate and combined treatment at the community level, the reinforcement of advice to health care providers, and accelerated referrals of severe cases [10, 13–15].

The gap between LLIN efficacy, high investment efforts toward its implementation and the effectiveness of interventions have been largely explored by social science researchers [16]. Moreover, qualitative research approaches have proven its place in malaria prevention and control strategies by “seeking to describe and understand what malaria means” rather than measuring indicators [17]. Some qualitative studies have aimed at analysing the impact of representations of malaria on prevention practices and care-seeking behaviours. In many settings, malaria is not considered a serious sickness and, therefore, adherence to both preventative tools and treatment remains a challenge [15, 16]. Factors such as cultural beliefs, risk perception, sleeping comfort or discomfort or sleeping space settings are known to influence LLIN utilization at the household and community levels [18–23]. Therefore, many socio-cultural factors have been highlighted and taken into account in communications and messaging through behaviour change strategies in many countries [16]. However, the influence of the policy of prioritizing malaria preventions and control strategies for under-fives on over-fives remains largely unexplored and, presumably, has not been considered in the policies to date. Regarding LLINs, beyond their irregular and decreasing use over time [24, 25], very few studies have explored the practices of use by children over 5 years of age. Among malaria prevention and control strategies, LLINs are specific in that their protective efficacy requires regular use, adherence to their use by the entire family, and accessibility [1]. The use of LLINs is intrinsically linked to people's perceptions of malaria, to their understanding of the modes of transmission, to the ecological, economic and climatic contexts, and, moreover, to the representations of LLINs or the perceived vulnerabilities of certain groups of individuals to malaria (e.g., pregnant women and young children) [15, 17]. Thus, the aim of this study was to analyse LLINs as a sociocultural object and to identify the sociocultural contexts in which children aged 5 to 15 years grow up, in view of the promotion of LLIN use under the framework of malaria control strategies and policies implemented in Madagascar, as well as the factors that may determine their use. Social vulnerability to malaria is considered here as the situation of a specific group of individuals within an environment of "social organizations, cultural norms and beliefs" that promotes "the development of the disease" and influences the "observed distribution" of malaria morbidity [17].

## Research setting

### Epidemiological profiles

**Fig. 1:**
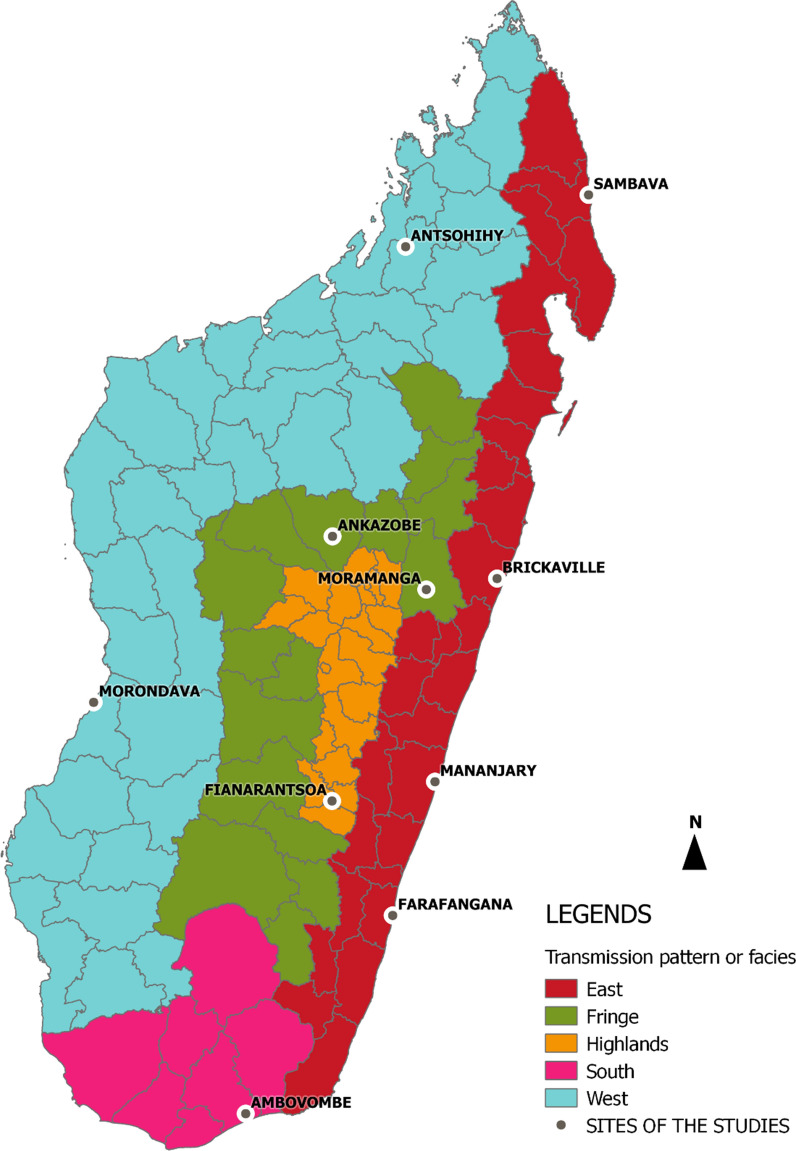
Malaria transmission patterns in the districts of Madagascar and sites of the three qualitative studies

Madagascar has 5 malaria epidemiological profiles, sometimes called '*facies*', which vary according to seasonality and transmission duration (Fig. 1). The equatorial profile, spread over the east coast, is characterized by a high level of perennial transmission where malaria is most prevalent. The tropical profile, on the west coast, has a transmission season lasting approximately six months, between October and April. These two coastal regions represent the highest endemic profiles. In the Central Highlands profile, malaria is unstable, episodic or epidemic between January and April. In the sub-desert profile in the south, transmission is episodic and of short duration. In the intermediate-altitude zone, called the margins, transmission is episodic from mid-November to May [26].

### The place of LLINs in the strategy to fight malaria in Madagascar

Madagascar's national malaria control strategy is based on the World Health Organization (WHO) technical strategy. Specifically, it occupies the three pillars of the strategy, namely, guaranteeing universal access to prevention, malaria care (diagnosis and treatment), and strengthening surveillance [27]. In this internationally oriented policy, children under 5 years of age are among the priority targets in the fight against malaria. In Madagascar, this priority is reflected in the free community management of uncomplicated malaria cases in these children, which is an integral part of the Integrated Management of Childhood Illness programme. Malaria is diagnosed via a rapid diagnostic test for children suffering from fever and positive cases are treated with artemisinin-based combination therapy by community health workers. In addition, the policy implements free routine distributions of LLINs in health centres during prenatal care, when children under 1 year old are vaccinated, and during consultations with children under 5 years old suffering from malaria [12, 15].

Apart from this targeted LLIN distribution channel, a mass distribution campaign, known as "the universal distribution strategy," has been conducted every 3 years (corresponding to the duration of LLIN effectiveness) since 2009. Routine mass distributions are carried out in highly endemic districts, comprising 108 of the 119 districts in Madagascar in 2018 [7].

Social marketing distribution is continuous and complementary to the other channels. It consists of supplying commercial businesses with LLINs at subsidized prices to meet the demands of households due to net deterioration, loss, or increases in household size [1, 26]. This type of intervention covers 21 out of Madagascar's 22 regions [26].

Communication about LLINs is inseparable from the evolution of free distribution policy and other malaria prevention and control strategies. From 2002 to 2006, the first routine distribution of LLINs took place in health centres and was exclusively aimed at pregnant women and children under 5 years of age. These original messages focused on the so-called "biologically vulnerable" groups through multiple channels: posters, audiovisual broadcasts, community approaches and mass sensitization as well as within the health centres [28]. At that time, indoor residual spraying was the predominant form of malaria prevention and control strategies [29]. Between 2007 and 2012, LLINs became a priority strategy in the malaria control policy: the objective was to distribute two LLINs per household [29]. In 2009, mass distribution was implemented for the first time in 91 highly endemic districts [29]. From 2012 to date, the objective of the universal distribution strategy has been revised; now, one LLIN is distributed for every two people. In addition, communication about LLINs has gradually moved away from the notion of vulnerable populations and instead embraced the principle of protecting "all household members under LLINs" [12, 30].

### Sociocultural context and territorial organization

Beyond its malaria epidemiological complexity, Madagascar is characterized by the diversity of its people. Eighteen ethnic groups constitute its population that differ in terms of where they live (e.g., central highlands, coastal regions, primary forests, and plains), the different resources at their disposal (in terms of agricultural areas, food resources, and mining resources) and sociocultural contexts (taboos, rites, food practices, and religious beliefs) [31].

The smallest administrative district in Madagascar is called a *fokontany*. In a rural configuration, a *fokontany* often includes several small hamlets or villages at varying distances from each other. The commune is a group of *fokontany* whose chief town usually hosts a market and a public health facility that provides access to primary health care, called the *Centre de Santé de Base*. Other private or confessional health facilities may be accessible at the commune level. One day a week is designated as a market day, which is well known to all the inhabitants in the surrounding area. On market day, the villagers gather not only to stock up on foodstuffs and goods for everyday use (salt, oil, matches, sugar, coffee) but also to sell agricultural or livestock products. Market day is a time for meeting and community effervescence; thus, the public primary health care centres capitalize on this opportunity to organize activities such as antenatal consultations, vaccinations and LLIN distribution. The majority of rural communes have roads, but they are often in extremely poor condition and not always accessible by vehicles during rainy seasons.

## Methods

This article is based on the results of 3 qualitative studies on malaria conducted between 2012 and 2016 in 10 districts of Madagascar by the Institut Pasteur de Madagascar (IPM) and the Institut de Recherche pour le Développement (IRD). The 10 study areas covered the 5 main malaria facies and 10 of the 18 ethnic groups. The following data collection tools were employed: (i) semistructured interviews with various categories of individuals (villagers and representatives of village authorities, health workers, traditional healers and traditional birth attendants), (ii) direct observations (e.g., living conditions, installation and use of mosquito nets, environment, water supply point, hygiene, queuing for consultation at the health centres, health worker availability) and (iii) informal interviews.

### Determinants of access to malaria control methods and their impact (MEDALI)—qualitative component—2012–2013

MEDALI is a study of the impact of the interventions deployed as part of the Global Fund and National Malaria Control Programme funding in Madagascar. Carried out between 2012 and 2013, this multidisciplinary study covers all of Madagascar's epidemiological profiles [32]. It includes a quantitative component focused on evaluating the effectiveness of malaria prevention and control strategies, complemented by sociodemographic and behavioural components. A second qualitative component (on which this article is based) aims to explore the sociobehavioural factors that interact with malaria control interventions, hindering or facilitating their effectiveness. Specifically, this section focused on the reasons for using health centres in cases of fever and the acceptance of the proposed malaria prevention and control strategies (LLINs and indoor residual spraying). Surveys by semidirective interviews and observations were conducted in 4 zones located in various epidemiological contexts: *Moramanga, Antsohihy, Fianarantsoa* and *Mananjary*. These districts cover the *Bezanozano, Tsimihety, Betsileo* and *Antambahoaka* ethnic groups. To ensure a diversified panel of health care utilization behaviours, the selected hamlets include both those that house the health centre and others more distant from it (between 1 and 1.5 h of travel time). Data collection was conducted from August to October 2012. A total of 70 semistructured interviews were conducted with 7 doctors/nurses, 8 community health workers, 3 traditional healers, 26 women and 26 men. The interviews explored three themes: perceptions and usual practices in case of fever (in adults and children); malaria prevention practices; knowledge and access to treatment. An article has already been published on the qualitative results of this study [15].

### PALEVALUT (operational evaluation of integrated malaria control)—anthropology of malaria control–2014

PALEVALUT is a multidisciplinary operational research programme, whose objective was to evaluate the effectiveness of malaria control strategies in real conditions. It aims to identify the factors that interfere with strategies, whether they are psychological, social, cultural, organizational or economic in nature. This large-scale programme, funded by the 5% Initiative, was implemented in 5 sub-Saharan African countries, including Madagascar, in 2014 [33–35]. The socio-anthropological component aims to analyse the social and cultural determinants of the use of control strategies (intrahousehold spraying and LLINs). The survey was carried out in the districts of *Brickaville* and *Ankazobe*. The choice of these two areas was guided by the diversity of implemented control strategies, the diversity of cultural (*Betsimisaraka* and *Merina/Betsileo* ethnic groups), linguistic, geographical and climatic contexts, and epidemiological profiles. Two communes per district were selected; within each selected commune, two *fokontany* were drawn at random: one close to the public primary health care centres and markets and the other more than 5 km away. The data collection, carried out in February and March 2014, included 58 semidirective interviews with 4 doctors/nurses, 7 community health workers, 3 traditional healers, 10 administrative and political leaders, 9 regional health officials and people from civil society who are actors in the fight against malaria: 16 women and 9 men. The themes investigated such concerns as popular representations of fever; popular discourse and perceptions of malaria prevention and control strategies; the involvement of biomedical care providers, health actors (community health workers and traditional healers), and political and administrative leaders active in the fight against malaria.

### Qualitative study on malaria: ownership and use of long-lasting insecticidal nets in Madagascar—2015

In light of the approximately 21 million LLINs distributed in Madagascar between 2005 and 2011 [12], USAID funded a qualitative study following the 2015 LLIN distribution in which the goal was to identify factors within households that affect net ownership and use. Four zones (*Ambovombe, Farafangana, Sambava* and *Morondava*) were selected: because of their diverse sociocultural contexts (*Antandroy, Antaifasy, Betsimisaraka* and *Sakalava* ethnic groups, respectively) and, in particular, the presence of factors likely to hinder LLIN use [36]. One *fokontany* per area was selected. The selection criterion was based on the effectiveness of LLIN distribution and the extent of malaria incidence in *fokontany*. Regarding the choice of households, the aim was to observe a diversified cross-section of LLIN practices throughout different life phases. Thus, based on census data, the study targeted nuclear families with a primiparous pregnant woman, families with at least one child under 5 years old, families with at least one child between 10 and 18 years old, and families whose children had already left the parental home. The data collection was carried out between March and June 2016, 6 to 10 months after the 2015 distribution campaign. A total of 64 semi-structured interviews and 64 direct observations of households benefiting from LLINs were conducted. The participatory data collection methodology, called Photovoice, was employed to capture images associated with the perceptions of the local population on malaria and LLINs [43]. Eight participants were equipped with cameras during the stay (4 men and 4 women) and given instructions to capture (four photos per participant) images associated with the following two questions: "In your opinion, what is malaria?" and "How can we protect ourselves against the disease transmitted by mosquitoes?" Focus groups were held with the participants to discuss their photo choices [37]. The topics covered in the individual interviews included malaria knowledge, its causes and prevention, images associated with LLINs, and the advantages, disadvantages, efficacy, risks; frequency, reasons and modalities of LLIN use. In addition, the prioritization and spatial organization of sleeping spaces was documented through direct observations, and constraints on the installation and maintenance of LLINs and the use of LLINs for other purposes were noted. Sleeping spaces are defined in this paper as spaces within dwellings dedicated to sleeping places for members of the household.

### Analyses

The 3 studies were conducted using the same analysis methodology, comprising a total of 192 semi-structured interviews. For the primary analysis, all the interviews were recorded, transcribed and translated into French. They were then subjected to a thematic analysis using analysis grids designed for each category of persons surveyed. This method made it possible to highlight recurrences and divergences in the participants' discourses according to the themes addressed. The analysis of these recurrences and divergences forms the basis of the results presented in the reports of the three studies.

For the secondary analyses, these report results were, in turn, analysed by following the same principles of thematic analysis. The aggregated analysis of these three studies was used to provide a holistic sociocultural description in terms of epidemiological profiles, ethnic groups and age groups beyond 5 years of age. The results were compiled by theme and sub-themes in an analysis grid in Excel: popular representations of LLIN, beliefs and rites related to sleeping space organization, LLIN availability and use, and perceptions of the populations most vulnerable to malaria. The analyses looked for the influence of pre-2009 LLIN distributions and focused on pregnant women and children under 5 years of age for the pivotal period of 2012–2015, during which the free and universal LLIN distribution strategy was adopted [24, 28]. In addition, data from the quantitative components of MEDALI and PALEVALUT were used to enrich the discussion.

## Results

To emphasize the tight link between social perception and social vulnerability of children aged 5 to 15 to malaria in relation to LLIN, the first section of this part will be devoted to representations of the bed nets in the different study areas. These data and some other data, though not specific to the 5–15-year-olds, will provide contextual factors related to the complex socio-cultural processes leading to social (“Research setting” section) and domestic (“Methods” section) practices and organizations related to LLINs that affect children over 5 years old in Madagascar. In addition, global data on LLIN use were included to provide the reader a better understanding.

### Representations of bed nets: levers and obstacles

#### Mosquito net adoption: a "fombandrazana" or tradition in some areas

In 4 of the 10 areas, the use of mosquito nets is common practice (*Antsohihy, Mananjary, Farafangana, Sambava*); however, their use is not systematically linked to the fight against malaria. Historically, mosquito nets are valuable objects: older people report that their parents already slept under mosquito nets (*Antsohihy*). However, in earlier times, mosquito nets were mainly reserved for adults. The idea has often been evoked that during the colonial era, mosquito nets were perceived as a high-class object—a sign of wealth—and therefore appreciated by the population (*Antsohihy, Mananjary, Farafangana, Sambava*). The main reason for using the mosquito net was the comfort provided by the object during sleep: preventing nuisances caused by insects and preserving the couple's privacy. In *Mananjary* and *Farafangana*, nets are intrinsically part of the kits offered on the occasion of weddings or births; thus, it is part of the family tradition in these regions. Before the circulation of information about malaria, such nets were sewn and used for the prestige they conferred upon to newlyweds and future parents. At the birth of a child, the net was required to protect the child from insect bites. This habit has been maintained through the present day, making marriage or childbirth key moments in the mosquito net use. Participants typically date their first use of nets to the time of their marriage. Even after the introduction of free LLIN distribution, the tradition of providing nets to newlyweds has continued, but the traditional bed net has been replaced by the LLIN. Today, free net distributions have democratized access to nets: wealth level no longer affects whether people have a net.

#### When the bed net provides information about the marital status of its user in the East Coast region

In community representations, the custom of including the net in the 'wedding trousseau' (*Mananjary* and *Farafangana*) led to an association between net use and marital status. Even today, the use of LLINs is a symbol of this status.

*“The use of mosquito nets is truly a tradition for us. Our ancestors used them. We can only be delighted that you have decided to give us free nets. It truly is a tradition for us. When we get married, we must have a mosquito net”* [Man, 40, *Farafangana*].

#### The mosquito net represents the idea of death: highlands and western region

In the highlands (*Moramanga, Ankazobe*) and the west coast (*Morondava*), people install the bodies of deceased under mosquito nets during the 3 days of the funeral rites to avoid contact between the body and environmental elements (insects were mentioned, especially flies, which participate in the decomposition of the body). This painful event imprints a macabre image associated with the use of a mosquito net, sleeping under a net sparks fears of the anguish of dying. White mosquito nets are culturally used for the dead. Thus, many respondents in these areas expressed an aversion to nets of this colour.

*“Personally, the disadvantage of the mosquito net is that when you sleep inside one, you look like a corpse”* [Man, 32, *Morondava*].

#### LLINs as a medicine and means of protection

Despite having specific representations in different regions, the LLIN is generally perceived positively, and the fact that they are now free is highly appreciated. The insecticide with which LLINs are impregnated is commonly called *"fanafody"* or "medicine" in the different areas surveyed. This reference to medicine in speeches has a rather positive connotation and is used in its protective sense: bringing well-being and health. The insecticide is also considered to be effective at eliminating insects.

*“The mosquito net is one of the objects one should now own, such as mattresses, cushions, and kitchen utensils for the newlyweds (...) Before, it was just a fashion, now it's a medicine, it's impregnated. It's medicine for health”* [Woman, 52, *Mananjary*].

The messages of awareness disseminated around the LLIN distributions are known and easily recited by the majority of the interlocutors, in particular its role as a barrier against mosquito bites and the importance of sleeping under one, especially for pregnant women and infants. However, the link between mosquitoes and malaria or *"tazomoka*,*"* literally translated as mosquito fever, is not spontaneously evoked in the speeches (unless the link between mosquito and fever or disease was already suggested by the question). During the Photovoice, many participants captured their LLINs in response to the question "how can one protect against mosquito-borne disease," demonstrating that they were cognizant of the link between disease and LLINs. In addition, fever and malaria are widely confused, the word *"tazo"* is mostly used to refer to malaria, while the same word is used to describe a febrile state.

“*If we don't sleep under an insecticide-treated net, we will be bitten by mosquitoes and will get the tazomoka*” [Man, 40, *Farafangana*].

“*The reason I use a mosquito net is because it protects against mosquitoes; they can't get in. Because if a person gets bitten, it causes "tazo," doesn't it*?” [Woman, 56, *Ankazobe*].

### Social organizations and practices related to LLINs

#### LLINs: inconsistent use not always related to malaria protection

Attitudes towards the use of LLINs varied across the survey areas. This variability can be explained by the greater or lesser prevalence of mosquitoes and (to a lesser extent) by the importance of malaria in the area under consideration. The use of mosquito nets is always justified first and foremost by the discomfort caused by mosquitoes, and malaria prevention comes second. This order of priority was found in all 10 study areas.

“*Why sleep under a mosquito net? Because you won't be bothered by mosquitoes, you will be able to sleep peacefully. There are so many things that can disturb one at night, such as cockroaches and centipedes. Mosquito nets truly protect us from many insects”* [Man, 50*, Farafangana*].

As this excerpt indicates, LLINs are used to protect against insect-related nuisances. In addition to the presence of mosquitoes, it appears that many factors can promote or limit the use of mosquito nets, including temperature (heat, cold), wind, brightness, and privacy preservation. The number and characteristics of nets owned also influence their use. Attention is given to the fabric quality, size, shape, colour, odour, and mesh size. In general, mosquito nets are preferred to soft, fine clothes, which are rectangular in shape and large enough to cover groups of sleepers. These are usually blue or some other coloured (white is considered too messy or to refer to the representations described above), with fine mesh sizes. Intermittent use of LLINs is the rule and is specifically determined by the level of comfort or discomfort felt during its use.

#### A distribution based on the size of the household and not on its composition (age and gender of its members)

The data of the 3 studies showed that the LLIN distribution strategy and its implementation in the real world determine the final availability of LLINs in households. An insufficient number of LLINs for all family members leaves some beds uncovered by LLINs and leads to practices that often compromise LLIN use among children over five.

Appreciation for the free LLIN distribution policy differs from one area to another. In 6 of the 10 zones surveyed, both mass and continuous distributions seem to follow the Ministry's recommendations, and there were very few complaints about the distribution procedure. Instead, the complaints received are related to the inadequacy of the nets received—not because of an anomaly in the distribution but because the share per household is calculated on the number of members of the household and not on the number of beds. In fact, as discussed further in this study, starting with children of a certain age, a "fady" (forbidden action or taboo) governs the organization of sleeping arrangements within a household. Beginning at age six to eight years old, male and female siblings can no longer share the same sleeping space, which leads to an increase in the number of LLINs required to cover the household sleeping spaces. In 3 of the 10 zones (Antsohihy, Ambovombe and Sambava), the insufficient number of LLINs is exacerbated by the presence of LLIN distribution anomalies. In these zones, several respondents complained that they did not receive LLINs or received insufficient numbers of LLINs (less than the expected number of 2 LLINs per household). In Antsohihy, some households possessed 3 LLINs while several had not received any. In Ambovombe, among those who received some, the number never exceeded 2 even for households comprising 8 or 9 people. The community health workers explain this situation by shortage of LLIN stocks. Other considerations may have limited the distribution of LLINs, as illustrated by the following health workers’ verbatim reports.

“*The problem is that routine distribution was suspended ‘from above’ (from the District Health Service) on the pretext that the population received too many during the campaigns and that those given mosquito nets may be neglected*” [Physician, *Ankazobe*].

“*In any case, pregnant women, whether or not they receive a malaria prevention kit, it doesn't matter !! They already benefit from other offers. It's not just mosquito nets and IPT (intermittent preventive treatment)!! ... First, they always benefit from all kinds of awareness measures. Second, if they visit an HIV/AIDS testing center, they will be screened. They also benefit from syphilis screening*” [District Health Service Manager, *Brickaville*].

When LLIN is lacking in a household, resourceful practices are used to acquire them, such as asking neighbours who received more than one LLIN or buying them at the market. However, the data available did not permit to clarify the uncertainty about the type (LLIN or other) or number (sufficient or not for the family) of purchased LLINs, nor about the exact reasons for distribution failures.

#### Some practices particularly expose young people to malaria

In coastal areas (*Antsohihy, Mananjary, Brickaville, Farafangana, Sambava*), during periods of high heat (October to April), many people refuse to use mosquito nets because they are too hot. In addition, while the sleeping time usually spans from approximately 8 p.m. to 5 a.m., in the summer, people wait for the heat to drop and go to bed after 9 p.m. Sociability practices such as parties with friends and family discussions, specifically called *"débat"* in *Ambovombe*, extend this time of exposure to mosquitoes to an undefined length. However, during this time of year, mosquitoes are present in high densities. Thus, even people who protect themselves during sleep are still subject to mosquito bites because they are exposed longer before going to bed.

*"We always do it like this [she hits her arm with a cloth] we use this cloth to repel mosquitoes when we want to talk in the evening with the neighbours, [she mimes hitting herself] like this, so that the mosquitoes do not bite us. When we get ready to sleep, we put the net down and go to bed*” [Woman, 36, *Ambovombe*].

Very young children, who are perceived as vulnerable, are put under nets early on and are therefore better protected from exposure to mosquito bites. In contrast, children over five years of age and adolescents take advantage of this time to play outdoors (*Ambovombe, Antsohihy, Mananjary, Morondava, Farafangana*). Thus, this group of children runs a higher risk of mosquito bites.

### Domestic organizations and practices related to LLINs

#### Organization of the sleeping space

The characteristics of the houses control the organization of the domestic sleeping spaces. Although the study areas are mostly in rural areas (9/10), they differ in terms of spatial distribution and building materials. Everywhere, however, the houses tend to consist of a single living room (on average 9 m^2^), which is transformed into a sleeping area for the night. The kitchen and showers are located outside. This leads to a particular spatial distribution of sleeping spaces: if a bed is available, the parents and children under 5 years of age sleep in the bed under mosquito nets (A bed here is defined as a sleeping surface raised from the ground, whether it has a mattress or not, of any kind and regardless of its material of manufacture (wood, bamboo, metal, etc.) or quality of finish.). The rest of the family (those beyond this age) sleep on the floor or on a sofa, usually without a mosquito net (*Brickaville, Ankazobe, Ambovombe, Mananjary, Farafangana, Sambava*).

“*Here, they have only one room, and most of the time, each family has many children, some of whom sleep on the floor. Only those who sleep on the bed have a mosquito net and are protected; those on the floor do not*” [ Health worker, *Brickaville*].

When there is no bed in the sleeping space and everyone sleeps on the floor, the mosquito net can cover up to 5 people (*Ambovombe, Mananjary, Farafangana*). Because the houses are small, they do not allow large households to deploy enough nets so that each person can sleep two-to-a-net, as recommended. However, even without a bed, children up to 5 years old typically sleep with their parents.

#### The mother of the family, responsible for the LLIN

Mothers are responsible for the acquisition, installation and maintenance of LLINs in a household (purchasing, washing, designating who sleeps under a net). These tasks devolve to those tasked with tidying or furnishing the interior of the house and are generally part of a more global representation of the role of women in the home. Additionally, the deployment of mosquito nets before bedtime is the mother's responsibility, both for the couple's bed and for the children's bed. For this reason, some men in *Ankazobe*, for example, did not feel concerned about net awareness.

#### Prioritizing pregnant women and children under 5 years of age in the use of LLINs

In all the study zones, the vulnerability of pregnant women and children under 5 years of age to malaria is unanimously acknowledged by the different categories of respondents. This perception echoes the awareness messages on malaria. According to the villagers interviewed, the injunction: "It is necessary to sleep under a mosquito net" applies particularly to children under 5 years old and the pregnant women. Beyond the age of 5, some respondents say that children are no longer at risk of malaria (*Moramanga, Fianarantsoa*).

“*Our child always slept under a mosquito net until he was 5 years old. Beyond the age of five, there is no more risk of the tazomoka*” [Woman, 30, *Moramanga*].

Some households without children under 5 and pregnant women question the value of continuing to sleep under LLINs (*Mananjary, Antsohihy, Moramanga*). In 8 out of the 10 zones surveyed, it was observed that if a household has too few mosquito nets, the pregnant woman or the woman with the infant should benefit first, followed by parental couples with or without children under 5 years old, and last, by children over 5 years old. It seems that the head of the family is much more likely to sleep under LLINs than are children over 5 years of age. The man often sleeps where the woman does. The only exception to this rule is the *"mifana"* period in *Ambovombe* (the confinement period after childbirth), where for 3 months, the man cannot share the same bed (and therefore the net) with the mother and the newborn. Moreover, according to interviews recorded, malaria is considered less virulent for men than for women (*Farafangana*). Men are "resistant, immunized" against malaria, and men's skin is thicker and, therefore, impenetrable to mosquito bites (*Ambovombe, Farafangana, Sambava, Morondava*). In *Farafangana*, it was noted that men acquire their "immunity" through physical strength due to heavy work in the fields. Therefore, for groups perceived as not at risk, even the availability of a LLIN for all members of the household does not necessarily lead to their regular use.

#### A taboo on sharing the same sleeping space for children of the opposite sex

When a child reaches the age of 6, he or she leaves the parents' bed for another in the family home. The primary reason given for this separation is that by this age, children are old enough to no longer need to sleep under a mosquito net. Indeed, before this age, they are still perceived to be highly vulnerable to malaria; therefore, they must sleep with their parents under mosquito nets.

*“I am the one who sleeps under a net, even though I have three children, the last of whom is 6 years old. Because the children are already grown up, they do not sleep under a net. I was told at the hospital that pregnant women are the ones who should sleep under a net*” [Young woman, 26, Antsohihy].

From a certain age, a *"fady"* or taboo governs the distribution of beds within a household. Male and female siblings can no longer share the same sleeping space. Same-gender siblings, however, may share a sleeping space until one of them is married. The age at which the taboo begins to apply varies according to the zone: it is 8 years in *Antsohihy*, *Fianarantsoa* and *Moramanga*, and ranges from 10 to 13 years in *Ankazobe, Brickaville, Ambovombe, Farafangana, Manajary* and *Sambava*. A single mother can sleep with her son until he is 6 years old and with her daughter until she is 14 years old.

*“We have three mosquito nets and there are six of us. This is not enough, because some of my children are grown up and cannot sleep in the same bed because they are siblings*” [Man, 51, *Ankazobe*].

In *Ambovombe, Farafangana*, and *Mananjary*, this separation of children's beds sometimes means leaving the parental home. Indeed, the parents build a house for each child near the parental house and install them in it. In *Mananjary*, separation from the family home takes place at the age of 14 and is similar to an initiation to adult life. The separated children still share the family meal before they have found their "suitors." Once married, a girl moves to the home of her husband's family, while a young man will build a more suitable home not far from his parents' home. The LLIN is not being used during the entire period before marriage.

#### Refusal of unmarried young men to sleep under a net

In *Mananjary* and *Farafangana*, the association of the net with marriage, as described above, leads young unmarried men to avoid using LLINs. From the age of 10 until marriage, boys, who by then sleep outside the parental home, refuse to sleep under the net for fear of being considered married. The presence of a mosquito net in the home could signify to those around him that he has already acquired a wife.

*“Young unmarried men do not want to sleep under mosquito nets too much. They say it is married people who sleep under nets. Men who are not yet married and who are looking for a companion do not sleep under a mosquito net”* [Woman, 17, rural *Farafangana*].

Furthermore, the role attributed to women in the procurement, maintenance and installation of a mosquito net reinforces this perception. If a mosquito net is rolled out in a house, it is because a woman has installed it for the boy, thus inducing him to become part of a couple. In addition, men's sense of 'immunity' to malaria and resistance to mosquito bites reinforces the idea that the use of a net is not necessary.

According to one of the interlocutors, the association between net use and marriage is promulgated by his church and parallels "good fatherhood," which involves taking care of the family and putting a net in the home.

*“It is a man's role when he gets married to equip the house. After he considers himself an adult, he is no longer a child who does not care whether or not he sleeps under a net. The mosquito net is part of the necessary equipment for the house. At the church they explain to us that marriage is sacred, and as he becomes an adult, the man has to take care of the house. The mosquito net is one of those things to have. It used to be a matter of fashion to have a mosquito net. Now it is a medicine, it is for health*” [Man, 35-year-old *Mananjary*].

#### Fear of death prevents the use of LLINs

As described above, mosquito nets are equated with the death veil in the highland (*Moramanga* and *Ankazobe*) and western coastal (*Morondava*) regions. The fear of LLINs generated by this association of the object with the event of death deprives part of the community of LLIN protection. At least two population groups are victims of this fear: children old enough to understand the concept of death (approximately those over 5 years old) and the *Sakalava* ethnic groups, including their children.

“*The only problem [regarding the use of mosquito nets] is getting children into them. They get scared and escape from the bed (...) They equate the net with a death veil. However, this mostly happened the first time they slept under it; when we explained to them that it protects them against mosquitoes, they understood*” [Man, 48, *Ankazobe*].

“*Some people do not like to use the net because it makes them think they are dead. They still take the free mosquito nets, but then they sell them; they do not just want to sleep under them because they think it makes them look like corpses*” [Women, 45, *Morondava*].

## Discussion

### Decision-making process for LLIN recipients

The results of this study show that two independent, but primary additional factors contribute to the insufficient use of LLINs: anomalies in LLIN distribution (non-allocation or insufficient allocation) and the underestimation of sleeping spaces in the distributions due to taboos and the separated sleeping arrangements of siblings of opposite sexes. Responses to these LLIN inadequacies are all problematic because they leave part of the household without a LLIN, force people to ask their neighbours for one (which often simply transfers the problem to another household), or necessitate buying a net or LLIN at the market, which is limited by accessibility, especially financial accessibility, and introduces two uncertainties: the quality of the net (e.g., LLIN *versus* untreated net) and whether the number of nets bought is sufficient for the family. This insufficient number of LLINs in a household therefore forces it to choose which people should benefit from the sheltered area. Thus, a decision-making process takes place that affects registers of acquired knowledge and sociocultural logic. The information received during the awareness-raising efforts and during the LLIN acquisition, which repeatedly remind the population of the vulnerability of pregnant women and children under 5 years of age, has been adopted by the populations in all the study zones. The links established between married life and mosquito nets, which is found in most of the study areas, also has an effect on the decision-making process. For example, if the woman sleeps under a net, the man also sleeps under a net. Consequently, when there are insufficient numbers of LLINs, it is the children over 5 years old and young people not yet engaged in conjugal relationships who have the lowest priority for LLIN use.

### Exposure to mosquito bites of children over 5 years of age

The analysis of the 3 studies have demonstrated the social vulnerability of children over 5 years of age to malaria. They are more often exposed to mosquito bites because of insufficient LLIN use (as a consequence of representations, beliefs or priority given to night activities), the organization of household sleeping spaces and the prioritization of populations designated vulnerable by prevention programmes. In *Farafangana*, another recent IPM investigation found that children between 6 and 14 years old often do not sleep at their parents' home; instead, they play at night and often sleep together in a house called *"kidabo."* This house, which is built at the *fokontany* level, is not equipped with mosquito nets [38].

Kesteman et al*.* [11], as part of the quantitative study of the MEDALI project, found that 49.1% of under-fives had slept under a mosquito net the night before, but only 38.1% of 5-to-14-year-olds had slept under a net. The Malaria Indicator Survey (MIS) 2016 found a similar difference, although less striking (73.4% for under-fives vs 66.1% for children aged 5–14) [26]. The socio-epidemiological study PALEVALUT, in addition to bed net used, evaluated bedtimes and rising times (when one enters and leaves the LLIN) to define the notion of effective protection. The study found that 60.7% of under-fives were effectively protected *vs* 40.5% for 5–14-year-olds [39]. Despite variations from one study to another, the coverage of under-fives is consistently higher than for older children, which confirms the qualitative findings of these 3 studies.

These results were corroborated by a comparative study by Ricotta et al*.* [40] using data from 10 African countries between 2010 and 2013, where children aged 5–14 years are comprehensively the least protected against mosquitoes, often at the same rank (for Nigeria, Malawi, Rwanda, Madagascar, Mozambique and Uganda) or lower (Zimbabwe, Liberia) than men aged 15–49 years. Children under five years of age and pregnant women are invariably the first two groups of individuals who benefit from LLINs [40]. A recent analysis (2014–2015) of three countries, including Madagascar, Mali and Nigeria, reached a similar conclusion: children over five and adolescents are approximately twice as likely to sleep without a net as are children under five [41]. In Ethiopia, weekly longitudinal monitoring of LLIN use between 2009 and 2011 found a similar uniform situation over time—even after mass distribution of LLINs [31]. Similar results were found in a study in Indonesia, Timor-Leste [27]. As the actual study and that of Lam et al*.* [42] in Uganda illustrate, most populations understand the vulnerability of pregnant women and children under 5 years of age, which has been conveyed by previous policies through the LLIN distribution strategy and other communications. This information has had an impact on the choice of LLIN beneficiaries in households [42]. Children under 5 years of age benefit from LLINs, sometimes considerably more than pregnant women, but to the detriment of those over 5 years of age, especially when LLINs are more accessible [21, 24, 41–43].

### Epidemiological and immunological aspects of malaria in children over 5 years of age

Global data on the specific morbidity of children aged 5 to 15 years are scarce; the indicators are typically either aggregated for all ages or focused on children under 59 months (4 completed years) [44]. In Madagascar, the prevalence of *Plasmodium* carriage in this age group is significantly higher than that in children under 5 years of age [11]. Their greater exposure to mosquito bites could be a factor that explains this difference in prevalence. In the PALEVALUT study, which is representative of the general population, children between 5 and 15 years of age accounted for almost one third of the population (29.1%) and half (46.3%) of the global pool of gametocytes in the overall studied population (T. Kesteman, personal communication). Anti-gametocyte immunity is usually less developed among children than adults [45], suggesting that this age group may constitute a parasite reservoir that contributes to the perpetuation of malaria transmission in Madagascar [9, 46]. Consequently, malaria prevention in this population has a multiplicative effect on the rest of the population and deserves, at least in this respect, to be considered in public health policies, making a shift from malaria control to malaria elimination.

### Sociocultural factors in Madagascar and elsewhere

Cultural components as factors in net use have been found in all countries [22, 47, 48]. In Loreto, Peru, mosquito net use is integrated into people's habits. Long before the advent of LLINs, villagers slept under nets from a very young age, regardless of the season or heat [28]. However, the role women play in acquiring, installing and making decisions regarding which people sleep under LLINs differs from country to country. For example, in Nigeria and Mali, women play an important role in these responsibilities [41], while in Timor-Leste, it is the men, as the "heads of household," who decide who sleeps in which bed and who should receive protection [21]. Three of the actual study results have not yet been found in the literature from other countries: the association of marital status with net use, the allusion to death when sleeping under a net, and the taboo of siblings of opposite sexes sleeping in the same bed after a certain age. These last two representations were found in the south central region of Madagascar in *Ihorombe* in another qualitative study conducted by PSI Madagascar [49]. In addition, that study found that in this region, the gesture of lifting the mosquito net, knees bent, spouse in front (because the woman often gets up before the man in the morning to prepare the meal) represents a request for forgiveness towards her spouse and thus humiliates the men for no reason. Each time he gets out of the net, it is as if he is endlessly apologizing to his wife. This gesture, known as *"Mifaly vady,"* prevents some couples in this region from using LLINs [49]. These barriers to the use of LLINs in Madagascar seem to be region specific. Again, the recommendation to specify a control policy according to the sociocultural context of each geographical intervention area is unavoidable [17], including at subnational levels.

### The reasons for using LLINs and the risks involved

This study results confirm that comfort and discomfort factors are the primary reasons for the use or non-use of mosquito nets, as widely described in the literature. Heat is by far the primary cause of non-use in at least 20 studies, both quantitative and qualitative, including the studies in the review by Pulford et al*.* [18–20]. A few studies also cited mosquito discomfort as the primary factor favouring LLIN use [21–23]. In all these studies, these factors consistently resulted in intermittent LLIN use. Longer durations of exposure to mosquitoes due to a number of factors, such as heat or customs of talking at night outdoors, are similar to those in other countries [22, 50]. However, entomological data from PALEVALUT has shown that the bites of the mosquitoes responsible for malaria in the 2 study sites, *Brickaville* and *Ankazobe*, are 2 to 6 times more intense outdoors than indoors. Intense bite activity was recorded between 7 and 9 p.m. in *Ankazobe* and 3–6 a.m. in *Brickaville* [51]. In two studies in Uganda and Tanzania, the habit established by "previous positive experiences" of sleeping peacefully was cited as a factor in the development of a "net culture" according to Koenker et al*.* [18, 48] and has resulted in "consistent use of LLINs despite fluctuations in risk perception" [18]. In a nutshell, the main reasons for using LLINs were not directly linked with malaria prevention.

### Sleeping space is a real concern for children over 5 years of age

With regard to the domestic sleeping space and its organization, this study found that parents sleep together with children under 5 years of age, while other children sleep elsewhere without LLINs and usually without a bed. The results of the MEDALI sociodemographic study below provide a more precise perspective on the findings of this study [52]. An average family consists of 5.16 people, who possess 1.87 LLINs, live in 2.39 rooms, and sleep by 2.23 people. Children under the age of 14 constitute half (47%) of the population [52]. Iwashita et al. [23] monitored the sleeping arrangements of 95 rural dwellings in Kenya in detail and found that LLIN use is very low among children aged 5–14 years. Most interestingly, Iwashita et al*.* [23] suggested that this finding is due more to sleeping-space arrangements rather than to the specific prioritization of pregnant women and children under 5 years of age. Many older children sleep without a bed in living rooms and rooms with multiple beds because the infants sleep with their parents, who have priority for bed occupancy in the bedrooms. They showed that the most suitable place to hang a LLIN is on a bed and in a bedroom. For a person sleeping in another room or without a bed (on a couch or on the floor), the LLIN must be set up before sleeping; thus, the attachment is temporary and often difficult to put in place. In the morning, the sleeping place is repurposed for other uses (e.g., sofa to sit on, kitchen to cook) and, therefore, the net must be taken down, put away, and reinstalled again every evening [23]. The sleeping space configuration in this study appears to be more spacious than ours (16.7 m^2^ vs*.* 9 m^2^), with fewer family members (4.1 vs*.* 5.16), fewer bedrooms per dwelling (1.7 vs. 2.39), fewer beds per similar house (0.9 vs. 1.31) and more LLINs available per household (3.0 vs*.* 1.87). This comparison suggests an even more dramatic situation in the actual study settings regarding the exposure of children over 5 years of age due to little available space, more bedrooms, more people and fewer LLINs.

## Conclusions

The qualitative studies analysed in this paper found that perceptions, social and domestic practices around LLINs, and living conditions determine how children over 5 years of age used LLINs. Children over 5 are less protected against mosquito bites than are children under five because the population seems to have integrated knowledge concerning the vulnerability of younger children and prioritize this last one to sleep under LLIN. In addition, regional representations of mosquito nets contribute to the lower use of LLIN among children over 5: these include an association between married status and net use, allusions to death when sleeping under a net, and taboos regarding siblings of the opposite sex sleeping on the same bed after a certain age. The often-cited lack of LLINs in households combines with the prioritization of under-fives and the organization of domestic sleeping spaces, which results in older children sleeping outside in unprotected areas. Decision makers are invited to become aware of the social determinants of LLIN use documented across these three studies and the implications of lower LLIN use in 5–15-year-olds on the exposure to mosquito bites for these children. This increases the vulnerability of these children to malaria and decision makers need to reinforce the efforts already undertaken in this direction in Madagascar. Specifically, sociocultural factors related to the use of LLINs should be considered, and that policy should vary, adapting strategies and awareness-raising efforts to health policy makers and public health programme managers depending on the geographical areas concerned. In Madagascar, the number of LLINs distributed per household should be based on the number of beds rather than on the number of people, because factors such as the sex and age of children influence the organization of sleeping spaces and affect their probability of sleeping under a mosquito net.

## Data Availability

The data included in this study are taken from in-depth individual interviews and direct observations. Considering its collaborative nature, the data gathered are the property of the research institutions and are held in trust to protect the interests of the people studied. The data are not publicly available due to the content that could compromise the research participants’ privacy and confidentiality. The data that support the findings of this paper may be available from the authors upon reasonable request and with permission of the IPM and IRD.

## Abbreviations

IRD: Institut de Recherche pour le Développement
IPM: Institut Pasteur de Madagascar
LLIN: Long-lasting insecticidal net
PSI: Population Services International
RISE: Research, Innovation, Surveillance and Evaluation programme
